# Intrathorakale Unterdrucktherapie des Pleuraempyems unter Einsatz einer offenporigen Drainagefolie

**DOI:** 10.1007/s00104-022-01800-x

**Published:** 2023-02-08

**Authors:** Viktoria Betz, Vera van Ackeren, Ernst Scharsack, Bettina Stark, Christian Theodor Müller, Gunnar Loske

**Affiliations:** Klinik für Allgemein-, Viszeral-, Thorax- und Gefäßchirurgie, Katholisches Marienkrankenhaus gGmbH, Alfredstr. 9, 22087 Hamburg, Deutschland

**Keywords:** Ersteingriff, Komplikationen, Drainageelemente, Polyurethanschaum, Verbandswechsel, Primary intervention, Complications, Drainage element, Polyurethane foam, Dressing change

## Abstract

**Einleitung:**

Anhand einer Fallserie berichten wir über unsere ersten Erfahrungen mit einer intrathorakalen Unterdrucktherapie (ITNPT) in der stadienadaptierten Therapie des Pleuraempyems (PE).

**Material und Methoden:**

Die ITNPT ist eine Weiterentwicklung der Unterdrucktherapie für die intrathorakale Anwendung. Nach thoraxchirurgischem offenen Débridement wurde ein intrathorakaler Unterdruckverband installiert. Als Drainageelemente verwendeten wir eine dünne offenporige doppellagige Drainagefolie (OF) mit offenporigen Polyurethanschäumen (PUS). Ausschließlich die OF wurde in direktem Kontakt zum Lungenparenchym angelegt. Die Unterdruckerzeugung erfolgte mit einer elektronischen Pumpe (kontinuierlicher Sog, −75 mm Hg). In der Revisionthorakotomie wurde je nach Lokalbefund die ITNPT beendet oder fortgeführt.

**Ergebnisse:**

Es wurden 31 Patienten im PE-Stadium II und III behandelt. Die ITNPT erfolgte bereits beim Primäreingriff (*n* = 17) oder bei Revision (*n* = 14). Die ITNPT erfolgte über einen Dauer von *m* = 10 Tagen (2–18 Tage), Wechselintervall *m* = 4 d (2–6 d). Die intrathorakaler Unterdruckverband-Anlage wurde in *m* = 3,5 (1–6) mal vorgenommen.

Die Empyemhöhle verkleinerte und reinigte sich unter dem Sog kontinuierlich. Die OF hat ein minimales Eigenvolumen bei maximaler Resorptionsoberfläche. Nach Anlage des Unterdrucks besteht kein intrathorakales Totvolumen, das Parenchym kann sich entfalten.

**Diskussion:**

Die schonenden Materialeigenschaften der OF ermöglichen die ITNPT zur Behandlung des Pleuraempyems. Es ist eine gezielte lokale intrathorakale Sanierung des septischen Focus in Ergänzung zur operativen Therapie möglich. Das Behandlungsregime erfordert wiederholte operative Verbandswechsel. Die Methode ist geeignet zur Behandlung komplizierter PIeuraempyeme im Stadium II und III.

**Konklusion:**

Die OF kann als intrathorakales Drainageelement zur ITNPT bei Pleuraempyemen verwendet werden. Das Indikationsspektrum der Unterdrucktherapie erweitert sich um diese neue Anwendungsoption.

## Abstract (en)

**Introduction:**

We report our initial experience with intrathoracic negative pressure therapy (ITNPT) in the stage-adjusted therapy of pleural empyema (PE) based on a case series.

**Materials and Methods:**

ITNPT represents a further development in negative pressure therapy that is designed to be used in the thoracic cavity. After thoracic surgical open debridement, an intrathoracic negative pressure dressing was inserted. The drainage elements used were a thin open-pore double-layer drainage film (OF) with open-pore polyurethane foams (PUF). Only the OF was placed in direct contact with the lung parenchyma. Negative pressure was generated using an electronic pump (continuous suction, −75 mm Hg). In revision thoracotomies, ITNPT was stopped or continued depending on local findings.

**Results:**

31 patients with stage II and III pleural empyemas were treated. ITNPT was administered at the time of primary procedure (*n* = 17) or revision (*n* = 14). ITNPT was given over a duration of *m* = 10 days (2–18 days), change interval *m* = 4 d (2–6 d). The application of intrathoracic negative pressure dressings was performed *m* = 3.5 (1–6) times.

The empyema cavity continuously reduced in size and was cleansed by the suction. The OF has a minimum intrinsic volume with maximum absorption surface. Once negative pressure is established, there is no intrathoracic dead volume and the parenchyma can expand.

**Discussion:**

The protective material properties of OF make ITNPT suitable for the treatment of pleural empyema. Targeted local intrathoracic drainage of the septic focus is a possible adjunct to surgery. The treatment regimen requires surgical dressings to be changed repeatedly. The method is suitable for the treatment of complex stage II and III pleural empyemas.

**Conclusion:**

The OF can be used as an intrathoracic drainage element for ITNPT in pleural empyema. This new application option expands the range of indications for negative pressure therapy.

## Hintergrund

Das Pleuraempyem (PE) ist eine schwerwiegende Erkrankung, die häufig der thoraxchirurgischen Therapie zugeführt werden muss. Die Inzidenz nimmt weltweit mit einer Mortalität bis zu 20 % zu [5, 24]. Bei einem PE handelt es sich um eine bakterielle Infektion des Pleuraspaltes mit Beteiligung beider Pleurablätter. Das PE kann parapneumonisch, postoperativ nach Lungeneingriffen sowie auch bei anderen von der Lunge ausgehenden Erkrankungen, wie z. B. Bronchiektasen, auftreten [6, 21, 22]. Auch eine extrapleural gelegene Keimverschleppung, z. B. von intraabdominal, kann ursächlich sein.

Es werden gemäß der American Thoracic Society nach dem Krankheitsverlauf drei Stadien des PE unterschieden. Die Therapie richtet sich nach dem jeweiligen Stadium der Erkrankung [26, 28]. Sie sollte sich auf die Kontrolle des Infektfokus, auf die Entlastung von Sekreten und putriden Verhalten sowie auf die Reexpansion des Lungengewebes und die Wiedererlangung einer physiologischen respiratorischen Mobilität des Brustkorbs konzentrieren [23]. Supportiv erfolgt in allen Stadien eine parenterale Antibiotikagabe in Anlehnung an die Ursache der Infektion (ambulant erworben, nosokomial) [9, 23].

Im **Stadium I (exsudative Phase)** beim Vorliegen eines meist sterilen Ergusses ohne ausgeprägte Septenbildung sowie mit Pleuraverdickung erfolgt therapeutisch die Anlage einer Thoraxdrainage mit zunächst kalkulierter und später gezielter Antibiotikatherapie [1, 8, 25, 26].

Das **Stadium II (fibrinopurulente Phase)** ist gekennzeichnet durch die Eindickung des Sekretes mit Bildung dicker Fibrinbeläge und Membranen sowie durch eine Eiterbildung im Pleuraspalt mit Gefahr einer Sepsis [1, 25, 29]. Therapeutisch wird, vorzugsweise per videoassistierter Thorakoskopie (VATS), ein Débridement und ggf. eine Dekortikation durchgeführt [8, 24, 26].

Im **Stadium III (Organisationsphase) **kann das PE in eine chronische Form mit Verwachsung der Pleurablätter, Schwartenbildung sowie Fesselung des Lungengewebes übergehen [1, 25, 29]. Therapeutisch ist eine Thorakotomie mit offen-chirurgischer Dekortikation erforderlich [10, 23, 26, 28]. Zudem wurde auch im Stadium III in den letzten Jahren eine thorakoskopische Herangehensweise per VATS versucht [11, 23].

Der Einsatz der Unterdrucktherapie im Rahmen der Wundbehandlung oberflächlicher oder tiefer, an schwierigen Körperstellen gelegener, sekundär heilender Wunden ist weit verbreitet. Offenporige Polyurethanschäume (PUS) werden in die Wunde eingelegt und mit einer Klebefolie versiegelt. Mittels einer Schlauchverbindung wird mit einer elektronischen Pumpe ein Unterdruck angelegt. An der Körperoberfläche wird die Unterdrucktherapie auch zum temporären Bauchdeckenverschluss und bei größeren Substanzdefekten z. B. der Bauch- oder Thoraxwand genutzt [3, 4].

Eine weitere wichtige Indikation der Unterdrucktherapie besteht in einem abdominellen Einsatz im Rahmen der Peritonitistherapie. Eine neuere Weiterentwicklung ist der intrakorporale Einsatz im postoperativen Komplikationsmanagement bei Anastomoseninsuffizienzen des oberen und unteren Gastrointestinaltraktes als endoskopische Unterdrucktherapie [13, 15–19].

Anhand einer Fallserie von 31 Patienten stellen wir unsere ersten Erfahrungen zur Behandlung des PE mit der intrathorakalen Unterdrucktherapie („intrathoracic negative pressure therapy“, ITNPT) unter Verwendung einer offenporigen Drainagefolie vor.

## Patienten

Einschlusskriterien waren ein computertomographisch oder per punctionem gesichertes Pleuraempyem im fortgeschrittenen Stadium II und III infolge einer Pneumonie (*n* = 14), bei Reempyem (*n* = 2) oder Lungenabszess (*n* = 5) sowie komplizierte Verläufe nach Ersteingriff, wie nach Pneumonektomie im Vorfeld (*n* = 5), nach Lungenteilresektion im Rahmen einer Tumorerkrankung (*n* = 2) sowie nach Ösophagusperforation bzw. -resektion (*n* = 1); bei 2 Patienten lag ein Empyema necessitatis vor (*n* = 2).

Die Patienten wurden vor den operativen Eingriffen hinsichtlich einer intraoperativ ggf. erforderlichen Anlage einer ITNPT mündlich und schriftlich aufgeklärt.

## Material und Methoden

Die Unterdrucktherapie hat sich innerhalb weniger Jahre als wichtige Therapiemaßnahme etablieren können [3, 17]. Die ITNPT beschreibt die Weiterentwicklung für die intrathorakale Anwendung. Das technische Wirkprinzip der Unterdrucktherapie („negative pressure therapy“, NPT) ist in allen Einsatzorten ähnlich.

Die benötigten technischen Voraussetzungen sind:

- ein Kompartiment, in dem ein Unterdruck aufgebaut werden kann,
- ein unterdruckerzeugendes System, z. B. eine elektronische Pumpe, sowie das mit diesem über Schläuche in Verbindung stehende
- sogvermittelnde, offenporige Drainageelement (DE).

Entlang der Oberfläche des offenporigen DE wird der therapeutische Unterdruck an die zu behandelnde Oberfläche angelegt.

### Kompartiment

Die NPT benötigt ein abgeschlossenes, luftdichtes Kompartiment. Bei der NPT an der Körperoberfläche wird dieses mittels okkludierender Folien geschaffen. Bei der Anwendung in der Abdominalhöhle muss diese nach operativer Eröffnung durch okkludierende Verbände und/oder operative Nähte luftdicht verschlossen werden, um einen Unterdruck zu installieren.

Analog gilt dieses auch für die ITNPT. Auch hier wird die Wunde nach dem thoraxchirurgischen Eingriff per Naht oder durch einen okkludierenden Verband verschlossen. Als Besonderheit benötigt die Pleurahöhle bereits physiologisch ein Unterdruckniveau für die Entfaltung des Lungenparenchyms.

### Unterdruckerzeugendes System

**Figure 1:**
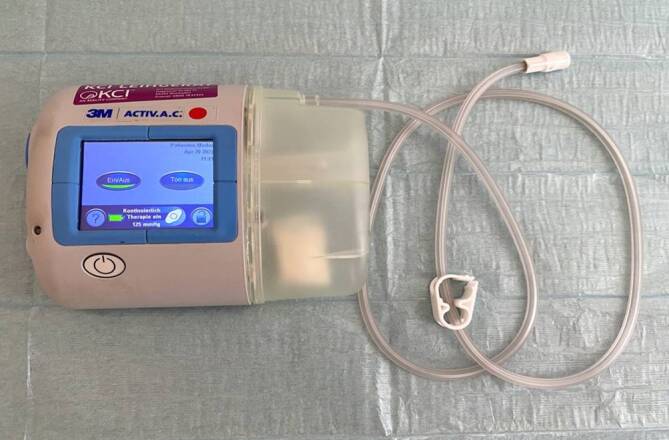

Für die Unterdruckerzeugung der ITNPT wurde eine elektronische Pumpe eingesetzt, die ursprünglich für die Therapie an der Körperoberfläche entwickelt wurde, aber auch in der abdominellen NPT und in der endoskopischen NPT verwendet wird (ACTIV.A.C.™ Therapy System, KCI USA, Inc., San Antonio, Texas, USA; Abb. 1). Standardmäßig wurde ein therapeutischer Unterdruck von −75 mmHg sowie ein kontinuierlicher Sogmodus angelegt.

### Offenporige Drainageelemente

Für die Sogvermittlung der ITNPT wurden zwei verschiedene Drainagematerialien verwendet:

- offenporige doppellagige Drainagefolie (OF),
- offenporiger Polyurethanschaum (PUS).

Beide vereint die physikalische Eigenschaft der Offenporigkeit. Alle Poren der Materialien stehen miteinander in einem flüssigkeits- und gasleitenden Kontakt. Wenn an einer Stelle des Materials ein Unterdruck angelegt wird, wird dieser dadurch auf die gesamte Oberfläche und von hier auf die Wundfläche vermittelt. Auch wenn einige der Poren verstopft sein sollten, wird über andere kommunizierende Öffnungen der Sog aufrechterhalten. Die beiden von uns verwendeten Materialien unterscheiden sich in ihren physikalischen Eigenschaften.

#### Offenporiger Polyurethanschaum

**Figure 2:**
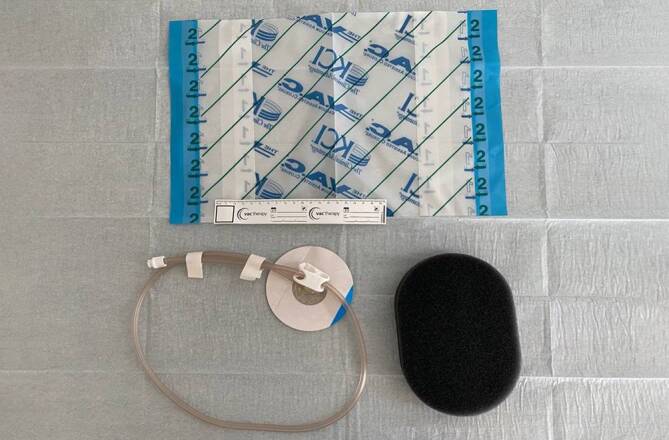

Der Schaumkörper des offenporigen, hydrophoben Polyurethanschaums (PUS; V.A.C. GRANUFOAM™ DRESSING, KCI USA, Inc., San Antonio, Texas, USA) hat ein relativ großes Volumen, welches unter dem Sog nur partiell kollabiert (Abb. 2). Die ungleichmäßigen Porenöffnungen (400–600 µm Größe) sind dicht benachbart und gehen ineinander über [27]. So können sie sich in Abhängigkeit von der Größe der Schaumoberfläche, der Porengröße und der Beschaffenheit der Wundfläche sehr fest auf den Wundgrund ansaugen und entsprechend stark anhaften.

Der PUS hat auf die Wundoberfläche stark débridierende Eigenschaften und lässt sich an Wunden der Körperoberfläche zur Wundreinigung und -konditionierung einsetzen.

Eine Kontraindikation ist die direkte Anwendung auf Peritoneum und Blutgefäßen.

Durch die Möglichkeit des innigen Wundkontaktes besteht ein relevantes Risiko für material- und sogbedingte Arrosionen. Als schwere Komplikationen wurden die Entstehung von transmuralen Darmfisteln sowie Blutungen beschrieben [2, 14].

#### Offenporige doppellagige Drainagefolie

**Figure 3:**
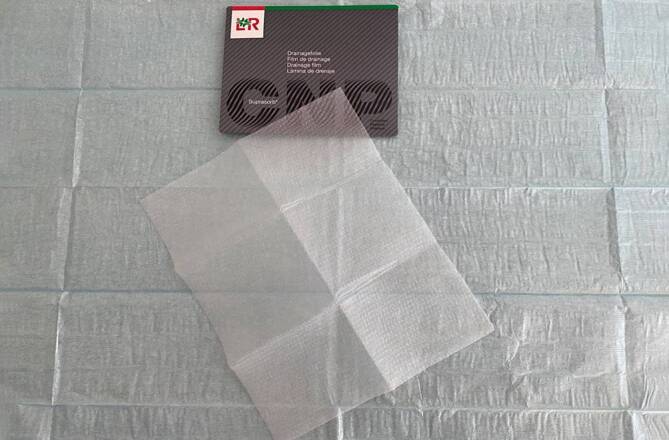

Bei der offenporigen doppellagigen Drainagefolie (OF; Suprasorb®CNP, Drainagefilm, Lohmann & Rauscher International GmbH & Co, Rengsdorf, Deutschland) handelt es sich um ein sehr dünnes Drainagematerial der Größe 77 × 60 cm bzw. 25 × 20 cm. Sie besteht aus zwei voneinander beabstandeten perforierten Membranen, deren Zwischenraum unter Sog nicht kollabiert (Abb. 3).

Flüssigkeiten und Gase können entlang der Folienblätter durch diese hindurch und in dem Zwischenraum offenporig drainiert werden. Der Unterdruck wird über die gesamte Folienoberfläche wirksam. Die Folie kollabiert auf Sog nicht. Sie hat ein minimales Volumen bei einer sehr großen Resorptionsfläche. Ein weiterer Unterschied zum PUS besteht in dem Abstand der Poren. Bei der OF sind gleichmäßig geformte Poren in einem regelmäßigen, brückenartigen engen Abstand zueinander angeordnet. Sie haftet nicht so fest wie ein PUS auf dem Gewebe. Die Débridementwirkung ist schwächer, aber schonender als beim PUS. Das Arrosionsrisiko auf vulnerablen Strukturen ist geringer. Im Gegensatz zum PUS kann die OF direkt auf Peritoneum gelegt werden. Durch ihre flexiblen Materialeigenschaften lässt sie sich gut über den Organen anmodellieren.

**Figure 4:**
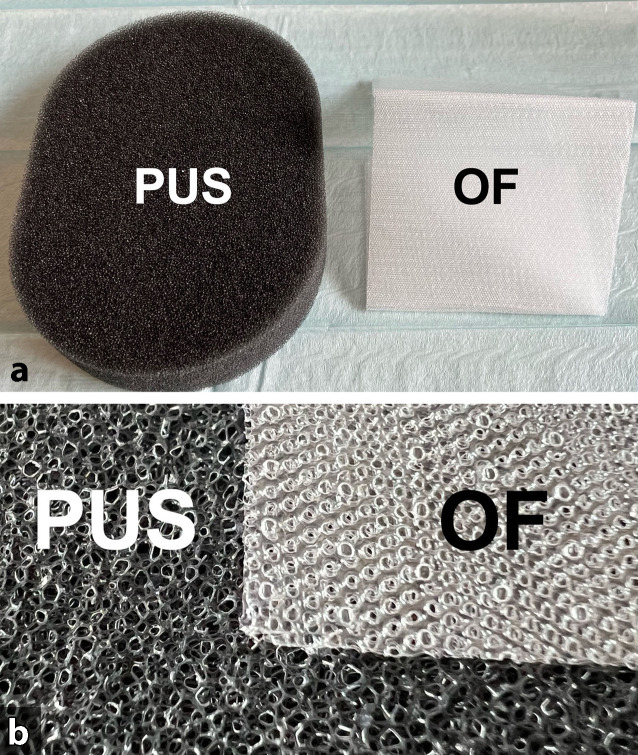

PUS und OF können komplementär verwendet werden (Abb. 4). PUS können mit der OF ummantelt werden (OFPUS). OFPUS haben die schonenden Oberflächeneigenschaften der OF.

In Abhängigkeit vom intrathorakalen Lokalbefund kamen bei unseren Patienten ausschließlich die OF und OFPUS zur intrathorakalen Anwendung: bei nicht ablösbaren Fibrinbelägen wurde die OF direkt auf das Lungenparenchym aufgebracht; bei lokalisierten Flüssigkeitskolliquationen legten wir einen OFPUS ein.

Im oberflächigen extrathorakalen Weichteilgewebe verwendeten wir sowohl PUS, OF und OFPUS.

### Operatives Vorgehen und Unterdruckverbandanlage

**Figure 5:**
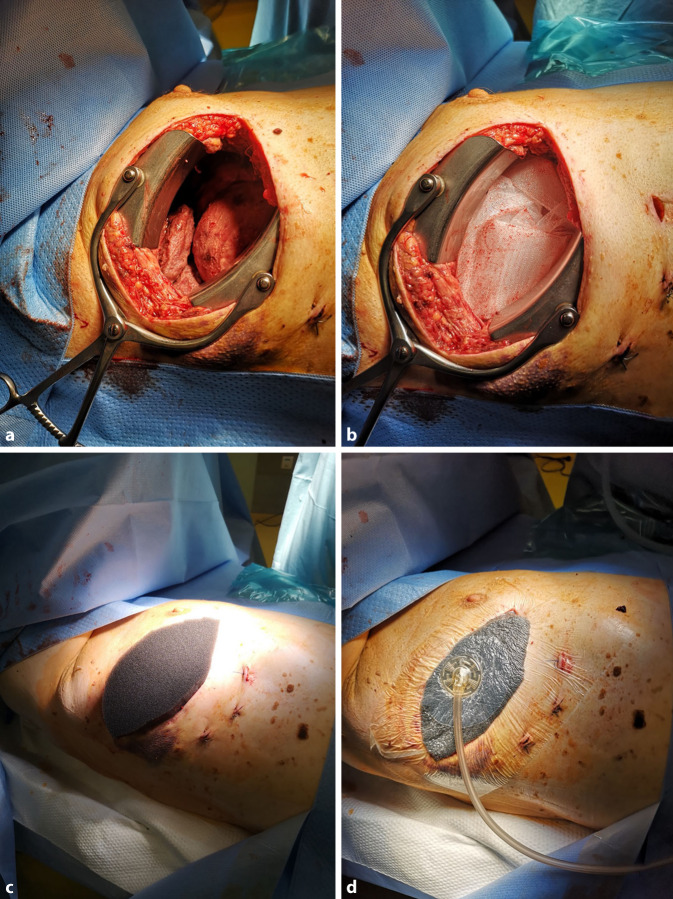

Operativ erfolgten nach anterolateraler Thorakotomie nach dem Eingehen in die Pleurahöhle in typischer Weise eine Dekortikation der beiden Pleurablätter sowie die Entfernung gekammerter Eiteransammlungen sowie fibrinöser Abszessmembranen (Abb. 5).

Nach ausgiebiger Spülung und Absaugung der Spülflüssigkeit wurde dann die OF der erforderlichen Größe zurechtgeschnitten, der Wundfläche angepasst und in die Pleurahöhle eingelegt. Es wurde versucht, das gesamte betroffene und entzündlich veränderte Lungenparenchym mit der großflächigen OF locker einzuscheiden, damit sich das Parenchym mit der es ummantelnden Folie entfalten konnte. Die OF befand sich somit im Pleuraspalt und wurde in die Thoraxwunde ausgeleitet. An der Öffnung der Thoraxwunde wurde die OF mit einem der Wundöffnung angepassten PUS in einen direkten Kontakt gebracht.

Die Wundränder wurden mit einem 3M™ Cavilon™ Hautschutzfilm versehen.

Die Thoraxwunde wurde nicht operativ verschlossen, sondern mit einer Okklusionsfolie versiegelt und hierüber dann das Trackpad zur Vakuumausübung installiert.

Nach Konnektierung mit der Pumpe wurde ein Unterdruck von −75 mm Hg an die Verbandsanordnung angelegt. PUS und die intrathorakal einliegende OF standen über den Materialkontakt in einer direkten unterdruckleitenden Verbindung.

Der Unterdruck wurde über die Kontaktsaugung der offenporigen Materialen nach intrathorakal geleitet.

### Verbandswechselintervall

Geplante Wechseleingriffe nach Beginn der Unterdrucktherapie wurden in Abhängigkeit der Ausprägung sowohl des intrathorakalen als auch des klinischen Befundes alle 2 bis 6 Tage durchgeführt. Dafür wurde das einliegende Unterdruckverbandsystem unter Operationsbedingungen entfernt, neuerliche Abstriche aus der Pleurahöhle entnommen, die Pleurahöhle ausgiebig gespült und die Spülflüssigkeit abgesaugt. Je nach Wundbefund erfolgte im Anschluss die neuerliche Anlage des Unterdruckverbandes in der beschriebenen Art und Weise.

## Ergebnisse

Von März 2017 bis Oktober 2021 wurden in der Klinik für Allgemein‑, Viszeral‑, Thorax- und Gefäßchirurgie des Marienkrankenhauses Hamburg gGmbH 216 Patienten mit einem Pleuraempyem versorgt. Bei 31 Patienten (14 %) wurde die operative Behandlung mit einer ITNPT vorgenommen (Tab. 1).

Die Patienten wurden uns aus anderen Fachabteilungen und Krankenhäusern nach dortiger Primärtherapie und bei z. T. lange vorbestehender Anamnese zur operativen Therapie zugewiesen.

Es wurden 20 männliche und 11 weibliche Patienten im Alter von 31 bis 85 Jahren behandelt.

Intraoperativ lagen PE der fortgeschrittenen Stadien II und III sowie komplizierte Verläufe nach Ersteingriff vor.

Bei 18 Patienten hatte eine Vorbehandlung stattgefunden; davon hatten 5 der Probanden eine Thoraxdrainage erhalten, 13 waren voroperiert.

Wir führten bei 30 Patienten eine anterolaterale Thorakotomie (THT) als Ersteingriff durch, wobei es sich bei 2 Patienten um eine Konversionsthorakotomie nach initialer VATS handelte; bei einem Patienten fand zunächst eine VATS statt mit im Verlauf dann erforderlicher Revisionsthorakotomie.

Bei 17 Patienten erfolgte aufgrund des vorliegenden schwerwiegenden Befundes die Einlage eines OFPUS bereits beim Ersteingriff; bei 14 Patienten wurde die OFPUS-Anlage aufgrund des im Verlauf nicht gebesserten Befundes bzw. als alternative Behandlungsmethode eingelegt.

Bei den übrigen Patienten wurde eine Unterdrucktherapie im Rahmen der aus anderen Gründen durchgeführten Revisionen erforderlich.

Gründe für die Revisionen waren ein Hämatothorax (*n* = 10), ein Chylothorax (*n* = 1), auftretende Reempyeme (*n* = 13), persistierende Parenchymfisteln mit Seropneumothorax und mit z. T. aufgetretenem Hautemphysem (*n* = 10), ein intraparenchymatöser Abszess mit notwendiger Keilresektion (*n* = 1), Wundheilungsstörungen der Thoraxwand (*n* = 9) mit Thoraxwandhämatom bzw. -abszess, eine aufgetretene Sepsis (*n* = 2) sowie eine spontan aufgetretene Ösophagusperforation mit -fistelung (*n* = 1).

Bei 4 Patienten kam es im Verlauf zu einer revisionspflichtigen Leckage des Unterdrucksystems.

Bei 5 Patienten wurde im Verlauf eine Omentumplastik durchgeführt; bei 4 Patienten wurde im Verlauf eine Thorakoplastik erforderlich. Bei einem Patienten musste ein Thorakostoma angelegt werden.

Fünf Patienten wurden im Rahmen der Langzeitbeatmung tracheotomiert.

Bei median 3,5 VAC-Anlagen (Range 1–6 Eingriffe) und einem medianen VAC-Wechselintervall von 4 Tagen (Range 2–6 Tage) lag die ITNPT-Behandlungsdauer bei median 10 Tagen (Range 2–18 Tage). Die Gesamtanzahl der erforderlichen operativen Eingriffe betrug median 8 Eingriffe (Range 2–16 Eingriffe).

Als Effekt der kontinuierlichen Sogwirkung konnte auf der Lungenoberfläche ein feinporiges regelmäßiges Ansaugmuster durch die Oberflächenstruktur der OF beobachtet werden. Das Parenchym wurde vom digitalen palpatorischen Eindruck weicher und bei der Luftinsufflation expandibler.

Bei jedem Verbandswechsel zeigten sich die Wundoberflächen zunehmend sauberer. Die Empyemhöhle bot unter der Therapie eine sichtbare Schrumpfung. Zudem konnte durch den kontinuierlichen Sog eine beginnende Granulation sowohl der Wundhöhle als auch des Subkutangewebes beobachtet werden.

Die Unterdruckverbände ließen sich pflegerisch gut versorgen; Hautmazerationen oder entzündliche Veränderungen durch die Verbandsanordnung wurden nicht beobachtet.

Der definitive Thoraxwandverschluss erfolgte nach entsprechender Besserung des Lokalbefundes nach Anwendung der NPT-Therapie unter Verwendung einer doppelschichtigen, offenporigen Folie bei 26 Patienten in herkömmlicher Weise unter Operationsbedingungen durch schichtweise Adaptation der Thoraxwandschichten mit anschließender Bülau-Drainageneinlage (2 Drainagen, gerade und gebogen, jeweils 28 Charrière).

### Komplikationen

Ein septischer Milzinfarkt mit erforderlicher Splenektomie, ein intraoperativ entstandener Zwerchfellriss, eine Hydronephrose mit erforderlicher DJ-Kathetereinlage sowie ein im Rahmen der septischen Situation aufgetretener entlastungswürdiger Abszess am Unterschenkelstumpf waren weitere während der Therapie beobachtete und behandlungsbedürftige Komplikationen, die nicht in direktem Zusammenhang mit der ITNPT standen.

Vier Patienten verstarben im Verlauf der stationären Behandlung; ursächlich waren bei allen Patienten ein septischer Schock, in Verbindung mit einer akuter Niereninsuffizienz (*n* = 2), einer septischen Kardiomyopathie bei septischer Streuung (*n* = 2) sowie einem Multiorganversagen (*n* = 1; Tab. 1).

**Table 1:** 

Patient	Alter	Geschlecht	Krankheitsbild	Vorbehandlungen (ja/nein)	Erstoperationsdatum	Ersteingriff	Anzahl VAC-Anlagen	Wechselintervalle (Tage)	Gesamtdauer VAC-Therapie (Tage)	Gesamtanzahl der Eingriffe	Therapiedauer gesamt (Tage)	Hospitaldauer Chirurgie (Tage)	Komplikationen	Tracheostoma	Tod (Ursache)
1	57	m	Parapneumonisches Empyem re mit Abszess ML	j (Drainage)	30.03.17	THT/Dekortikation	3	4	12	16	62	109	HämatoTx, Fistelung, Thorakoplastik, VAC-Leckage	j	n
2	74	m	Chronisches Empyem li Pneumonektomie bei BC 2013	j (Pneumektomie li bei BC 2013)	27.06.17	Re-THT Ii mit CNP-Folieneinlage	1	3	3	2	4	25	Omentumplastik	n	n
3	74	m	Chronisches Empyem li Pneumonektomie bei BC 2013	j (Pneumektomie li bei BC 2013)	04.12.17	THT, Teilthorakoplastik li mit CNP-Folieneinlage	2	4	8	3	8	20	Reempyem, Thorakoplastik mit Schwenklappen Lat	n	n
4	74	m	Chronisches Empyem li Pneumonektomie bei BC 2013	j (Pneumektomie li bei BC 2013)	04.01.18	Re-THT li, Dekortikation, CNP-Folieneinlage	3	5	15	4	14	20	Erweiterung der Thorakoplastik	n	n
5	74	m	Chronisches Empyem li Pneumonektomie bei BC 2013	j (Pneumektomie li bei BC 2013)	31.03.18	Re-THT, CNP-Folieneinlage	2	6	12	3	11	33	Thorakostoma	n	n
6	55	m	Parapneumonisches Empyem re	n	25.09.17	THT/Dekortikation	1	2	2	4	3	15	Revisionspflichtiger HämatoTx	n	j (sept. Schock, ANI, Kardiomyopathie)
7	55	m	Parapneumonisches Empyem bds. mit Abszess	n	30.03.18	THT/Abszessdrainage bds	1	4	4	5	35	112	Fistelung, Omentumplastik	j	n
8	64	w	Postpneumonischer Abszess LOL li	n	14.04.18	THT/LOL-Resektion li	1	6	6	3	16	77	Septischer Milzinfarkt; Splenektomie	j	j (septische Streuung, Kardiomyopathie, septischer Schock)
9	53	m	Empyema necessitatis li	n	25.05.18	THT/Dekortikation mit CNP-Folieneinlage li	1	4	4	2	5	23	Keine	n	n
10	45	m	Empyem li nach UL-Resektion bei „destroyed lung“/abszedierende Pneumonie	n	19.11.18	THT/LUL-Resektion li	3	5	15	12	38	162	HämatoTx, Reempyem, Wundheilungsstörung, Omentumplastik	n	n
11	85	w	Parapneumonisches Empyem III re	j (Drainage)	21.11.18	VATS/konv. THT/Dekortikation re	1	3	3	6	21	72	HämatoTx, Reempyem	n	n
12	65	m	Chronische kutane Fistelung/SerofibroTx li nach HITOC/Pneumonektomie	j (OP HITOC/Pneumonektomie li 09/18)	15.12.18	Re-THT, Fistelexzision, Ausräumung SerofibroTx, CNP-Folieneinlage li	6	3	18	7	18	82	HämatoTx, Empyem, Wundheilungsstörung, VAC-Leckage	n	n
13	66	m	Empyem und Abszess LOL li	j (THT li/Dekortikation/Lu-Abszessdrainage 10.05.19)	06.06.19	THT/Dekortikation/Abszessdrainage li	2	4	8	7	40	73	Reempyem, SeropneumoTx, Fistelung, Omentumplastik	j	j (septischer Schock, ANI)
14	31	m	Empyem und Abszess LOL re	j (Drainage)	29.01.20	VATS/Keilresektion/Dekortikation; THT im Verlauf re	3	3	9	7	20	61	Reempyem, Fistelung, Lu-Abszess, HämatoTx	n	n
15	82	w	Parapneumonisches Empyem re	n	24.02.20	THT/Dekortikation	1	4	4	3	15	30	HämatoTx	n	n
16	57	w	Reempyem li	j (THT/Dekortikation li 04/20)	12.06.20	THT/Dekortikation mit CNP-Folieneinlage li	1	3	3	2	4	13	Keine	n	n
17	79	m	Empyem re nach LOL-Resektion re bei BC	n	22.07.20	THT/LOL und ML-Resektion re	1	4	4	5	20	50	Revisionspflichtiger HämatoTx, Fistelung, Erguss, Hautemphysem	n	n
18	62	w	Reempyem und Abszess? LUL re	j (OP bei Empyem auswärtig)	06.10.20	THT/Dekortikation	1	2	2	4	10	27	VAC-Leckage	n	n
19	75	m	Parapneumonisches Empyem li	n	26.10.20	THT/Dekortikation li	1	3	3	6	21	38	Reempyem, Tx-Wandabszess?	n	n
20	55	m	Empyem III und Abszess? LUL re	n	02.11.20	THT/Dekortikation mit CNP-Folieneinlage re	1	2	2	5	12	103	Revisionspflichtiger HämatoTx, Reempyem	n	n
21	78	m	Parapneumonisches Empyem re	j (Drainage)	13.11.20	THT/Dekortikation mit CNP-Folieneinlage re	2	3	6	4	26	72	Intraoperativer Zwerchfellriss, persistierendes Empyem, ChyloTx, Thorakoplastik	n	n
22	59	m	Empyem bei Bronchusstumpfnekrose nach Resektion LOL li bei BC	j (VATS Keilresektion 08/20; LOL-Resektion li 10/20, CT-gesteuerte Punktion)	23.11.20	THT/Bronchusnachresektion/CNP-Folieneinlage	2	4	8	3	8	90	Fistelung, SeropneumoTx, Reempyem, Omentumplastik	n	n
23	70	w	Empyem li nach Osophagusperforation nach MIC-Fundoplicatio	j (MIC-Fundoplicatio auswärtig 03/21, LSK, Endosponge)	18.03.21	THT/Dekortikation li	1	4	4	7	79	99	Reempyem, Wundinfekt, Omentumplastik mit revisionspflichtiger Nachblutung, Ösophagektomie	J	n
24	68	w	Empyem III und Abszess LUL li	j (Drainage)	21.06.21	THT/Keilresektion/Dekortikation mit CNP-Folieneinlage li	2	4	8	5	23	28	Septischer Schock, Thoraxwandhämatom	n	n
25	64	w	Parapneumonisches Empyem li	n	24.06.21	THT/Dekortikation mit CNP-Folieneinlage li	2	4	8	3	8	24	Hydronephrose, DJ-Einlage	n	n
26	69	w	Parapneumisches Empyem re	n	06.07.21	VATS/konv. THT/Dekortikation re	5	3	15	8	31	44	Ösophagusfistel, Reempyem, Wundheilungsstörung, VAC-Leckage	n	n
27	70	w	Parapneumisches Empyem li	n	12.07.21	THT/Dekortikation mit CNP-Folieneinlage li	1	4	4	2	4	8	Wundheilungsstörung	n	n
28	74	m	Parapneumisches Empyem re	j (Ösophagusresektion auswärtig 15.06.21)	16.07.21	THT/Keilresektion/Dekortikation mit CNP-Folieneinlage	2	4	8	3	7	16	Fistelung, Wundheilungsstörung, Sepsis	n	j (septischer Schock, MOV)
29	85	w	Parapneumisches Empyem li	j (VATS/ Dekortikation li 08/21)	12.10.21	Re-THT Ii mit CNP-Folieneinlage	1	3	3	2	3	10	Reempyem	n	n
30	32	m	Postpneumonisches Empyem II re	j(VATS/Konv. THT/Dekortikation re 09/21)	19.10.21	Re-THT re, Dekortikation, CNP-Folieneinlage	1	3	3	2	3	17	Wundheilungsstörung	n	n
31	42	m	Empyema necessitatis li	n	12.10.21	THT/Dekortikation mit CNP-Folieneinlage li	2	4	8	7	17	33	Revisionspflichtiger HämatoTx, revisionspflichtiger Abszess li Fuß	n	n

*ANI* akute Niereninsuffizienz, *BC* Bronchialkarzinom, *DJ* Doppel‑J, *HITOC* hypertherme intrathorakale Chemotherapie, *j* ja, *li* links, *LOL* linker Oberlappen, *LUL* linker Unterlappen, *m* männlich, *MIC* minimal-invasive Chirurgie, *ML* Mittellappen, *MOV* Multiorganversagen, *n* nein, *re* rechts, *OP* Operation, *THT* Thorakotomie, *Tx* Thorax, *VAC* „vaccum assisted closure“, *VATS* videoassistierte Thorakoskopie, *w* weiblich

## Diskussion

Die Therapie des PE erfolgt stadienadaptiert nach den Richtlinien der American Thoracic Society [26, 28].

Im Idealfall gelingt es schon mit dem ersten Eingriff, endgültig die Situation zu beheben. Oft sind aber Revisionseingriffe erforderlich, um entstandenes Fibrin und Schwartengewebe zu entfernen und eine Fesselung der Lunge zu verhindern.

Bei der Unterdrucktherapie mit Polyurethanschäumen dürfen diese nicht direkt auf vulnerables Gewebe aufgelegt werden, da es durch die Anhaftung des Schaumes zu Gewebeverletzungen kommen kann. Im Bereich des Thorax ist die Unterdrucktherapie daher vor allem zur äußerlichen Wundbehandlung an der Körperoberfläche angewendet worden.

Nur wenige Berichte liegen zur intrathorakalen Anwendung der Unterdrucktherapie bei Pleuraempyemen bzw. Lungenabszessen vor [7, 12, 20–22, 28, 30]. Es wurden hierbei PUS mit einer darunterliegenden Schutzschicht, z. B. mittels mikroporöser Silikonfolie (Mepithel®), eingesetzt [7, 20].

Auf der Grundlage unserer langjährigen Erfahrungen mit der OF im endoskopischen und intraabdominellen Einsatz stellen wir mit dieser klinischen, retrospektiven, nichtrandomisierten Beobachtungsstudie die erste Fallserie zur intrathorakalen Anwendung der Unterdrucktherapie unter Verwendung einer doppellagigen, offenporigen Drainagefolie (OF) als neuen Therapieansatz vor. Wir zeigen, dass unter Verwendung dieses alternativen Drainagematerials die Unterdrucktherapie auch für die Pleurahöhle als neues Therapiekonzept für die Behandlung des Pleuraempyems entwickelt werden kann.

Die OF, welche in direktem Kontakt zu den intrathorakalen Organen angelegt wurde, wurde ursprünglich für den Einsatz in der Abdominalhöhle konzipiert. Anders als beim PUS erlauben die speziellen Materialeigenschaften der OF ihre direkte Installation zur Unterdruckausübung auch auf potenziell verletzlichem Gewebe, wie z. B. bei der intraabdominellen Behandlung von Peritonealorganen. Eine zusätzliche Abdeckung mit einer weiteren Schutzschicht ist nicht erforderlich.

Die dünne OF lässt sich sehr flexibel an das Lungenparenchym anmodellieren. Eine atemabhängige Expansion ist möglich. Einen deutlichen Vorteil der OF sehen wir in dem minimalen Eigenvolumen bei maximaler Resorptionsoberfläche. Der winzige Zwischenraum zwischen den beiden Folienblättern kollabiert auf Sog nicht. Der vollflächige Unterdruck wird hierdurch über die gesamte Folienoberfläche wirksam. Da die doppellagige Membrane sehr dünn ist, entsteht nach Soganlage durch das eingebrachte Material fast kein Totraumvolumen. Das Lungengewebe kann sich vollständig ausdehnen. Gleichzeitig schrumpft und reinigt sich die Empyemhöhle, die verbleibende Empyemresthöhle wird minimiert.

Ein wesentlicher Materialunterschied zum PUS besteht in dem Abstand der Poren zueinander. Bei der OF handelt es sich um gleichmäßig geformte Poren, die in einem regelmäßigen, brückenartigen engen Abstand zueinander angeordnet sind. Dieses Material haftet daher unter Sog weniger fest auf dem Gewebe als ein PUS. Das Arrosionsrisiko auf vulnerablen Strukturen ist geringer als beim PUS einzustufen. Insbesondere auf einem bereits granulierenden Wundbett kann sich ein PUS außerordentlich fest ansaugen. Diese Eigenschaft führt bei einer längeren Behandlungsdauer dazu, dass vulnerables Gewebe verletzt und arrodiert werden kann. Eine direkte Platzierung eines PUS auf peritonealen Organen oder Gefäßen ist daher nicht zulässig. Es besteht ein Risiko für Blutungen und Fistelentstehung. Wir zeigen in unserer Studie, dass die direkte Platzierung der OF mit Unterdruck auch auf pleuralem Gewebe möglich ist. Dennoch ist eine Entstehung von Fisteln potenziell denkbar, wurde aber in unserer Fallserie nicht beobachtet. Die Entfernung der OF war in allen Fällen schonend und ohne pleurale Verletzungssetzung möglich.

Insbesondere aus unseren zahlreichen klinischen Anwendungserfahrungen aus der endoskopischen und intraabdominellen Unterdrucktherapie wissen wir, dass die direkte Débridementwirkung der OF schwächer als beim PUS ausgeprägt ist. Bei der intrathorakalen Anwendung konnten wir beobachten, dass sich die inneren Wunden nach erfolgtem operativem Débridement mit dem OF-Verband kontinuierlich säuberten. Das mit der Folie in Kontakt stehende Gewebe zeigte eine flächige feinnoppige Granulationsoberfläche, die sich nach Beendigung der Therapie vollständig zurückbildete.

Der Vollständigkeit halber ist zu erwähnen, dass auch ein weißes Schwammmaterial aus Polyvinylalkohol (PVA) existiert, welches auf vulnerablem Gewebe eingesetzt werden kann (V.A.C. Whitefoam™ DRESSING, KCI USA, Inc., San Antonio, Texas, USA). Zum intrathorakalen Einsatz des Materials liegen uns keine eigenen Erfahrungen vor.

Die Unterdrucktherapie ist in der Lage, Flüssigkeiten optimal in aktiver Art und Weise zu drainieren. Infektiöse Sekrete werden durch die Sogwirkung eliminiert, die Keimbelastung reduziert und auch das entzündliche Gewebeödem lokal flächig drainiert, die lokale Perfusion wird verbessert. Als klinisches Zeichen hierfür und zum Vorteil der Lungenventilation und -perfusion palpiert sich das Lungenparenchym im Laufe der Behandlung zunehmend weicher und expandibler.

Analog zu unseren Erfahrungen mit der abdominellen und auch endoskopischen Unterdrucktherapie haben wir als unseren Standardunterdruck einen kontinuierlichen Sog von −75 mm Hg gewählt. Möglicherweise sind auch moderate Druckeinstellungen ebenso ausreichend. Diese Fragestellung könnte in weiteren Studien untersucht werden.

Die Gesamtanzahl der Revisionseingriffe bezogen auf die Anzahl der 31 Patienten erscheint zunächst hoch. Man muss hier in Relation setzen, dass es sich um ein Patientenkollektiv handelte, bei dem bereits ein komplizierter Verlauf vorlag bzw. aufgrund der Schwere des PE zu erwarten war. Die Revisionseingriffe lagen pathognomonisch dem Krankheitsbild des Pleuraempyems und dessen unterschiedlich schwerer Ausprägung im Patientengut zugrunde.

Die Vorstellung der Patienten erfolgte aus anderen Fachabteilungen und Krankenhäusern nach dortiger Primärtherapie und bei z. T. lange vorbestehender Anamnese in schon fortgeschrittenen Stadien.

Die Revisionseingriffe waren z. T. aufgrund schwerwiegender Komplikationen wie Hämatothorax, Chylothorax, Parenchymfisteln, Hautemphysem, intraparenchymatösem Abszess, Wundheilungsstörungen der Thoraxwand mit Thoraxwandhämatom bzw. -abszess, spontan aufgetretener Ösophagusperforation sowie einer aufgetretenen Sepsis erforderlich; diese Komplikationen standen nicht im Zusammenhang mit der Unterdrucktherapie, sondern traten bereits vor Beginn der ITNPT auf und induzierten sie bisweilen.

Aufgrund des selektierten Patientengutes mit zum Zeitpunkt der Indikationsstellung fortgeschrittener Empyemausprägung wurde die OF durch einen offen gewählten Operationszugang eingebracht.

Entsprechend unserem Dafürhalten wäre in keinem der besprochenen Fälle aufgrund der stark ausgeprägten Schwartenbildung eine VATS im Stadium III ausreichend gewesen; eine suffiziente Dekortikation wäre in diesen Fällen mit minimal-invasiven Instrumenten nicht erreichbar gewesen. Ein Vorgehen per VATS erschien uns daher aufgrund der in unserer Abteilung gemachten Erfahrungen in diesen Fällen nicht ausreichend Erfolg versprechend, da häufig in fortgeschrittenen Empyemstadien nach VATS mehr Revisionseingriffe erforderlich werden.

Ein thorakoskopischer Zugang würde ggf. eine kürzere Hospitalisationsdauer bedeuten; nachteilig wäre jedoch aufgrund der geringeren intraoperativen Übersicht im Vergleich zum offenen Zugang ein schwierigeres Einbringen und eine ggf. erschwerte und unvollständige Expansion der OF.

Letztlich waren in einem Drittel der Fälle noch Verplombungen im Sinne einer Omentumplastik oder Thorakoplastik erforderlich. Bei diesen Patienten kam es aufgrund vorbestehender intraparenchymatöser Abszesse mit Parenchymschrumpfung, nach erforderlichen Übernähungen bei persistierenden Parenchmyfisteln und nach z. B. Lobektomie zu Substanzdefekten, welche im Verlauf eine Auffüllung der Pleurahöhle mit Omentum majus bzw. bei nicht ausreichender Substanz dessen eine Thorakoplastik erforderlich machten. Auch dieses ist wiederum auf die initiale Empyemausprägung zurückzuführen und nicht als unzureichendes Behandlungsergebnis der Unterdrucktherapie zu werten.

Unter der Behandlung kam es bis auf einen Fall, in dem eine Thorakostomaanlage notwendig wurde, zu einer Ausheilung der Empyemerkrankung.

Die Letalitätsrate lag bei 12,9 % (4 von 31 Patienten).

Es ist anzumerken, dass es bei dem von uns gewählten operativen Vorgehen schon durch die Notwendigkeit des Wechsels des Verbandsmaterials zu einer erhöhten Rate an geplanten Revisionseingriffen kommt. Dieses könnte als ein berechtigter Nachteil der Methode angesehen werden. Vorstellbar ist, dass zukünftig bei weiterer Entwicklung der Methode und angepassten neuen Drainagematerialien in geeigneten Fällen auch minimal-invasive Techniken zum Einsatz kommen könnten.

Limitierende Faktoren der vorliegenden Arbeit sind die retrospektive Analyseform sowie das heterogene Patientengut. Die weitgehend standardisierte Vorgehensweise besteht ausschließlich in der intrathorakalen Verwendung der OF. Die Wundbehandlung und Verbandsanordnung müssen immer neu den vorliegenden individuellen Gegebenheiten angepasst und optimiert werden, um erfolgreich zu sein. Diese kreative Handhabung ist allerdings ein Kriterium, das sich in allen Anwendungsvarianten der Unterdrucktherapie wiederfindet. Die neuen Drainagematerialien bereichern die Möglichkeiten der Therapie erheblich.

Für die intrathorakale Therapie zugelassene Drainageprodukte oder Pumpen gibt es noch nicht.

Ein Nachteil könnte auch in der notwendigen Verbandsanlage unter Operationsbedingungen und Narkose gesehen werden. Im Prinzip entspricht dieses Vorgehen analog dem der Relaparotomie in der abdominellen Chirurgie.

## Fazit

In unserer Studie stellen wir die intrathorakale Unterdrucktherapie (ITNPT) als eine neue ergänzende Therapieoption bei der operativen Behandlung des komplizierten Pleuraempyems im Stadium II und III sowie bei komplizierten Verläufen nach Ersteingriff vor. Als innovatives Drainagematerial kam zusammen mit herkömmlichen, offenporigen Polyurethanschäumen eine dünne, doppellagige, offenporige Drainagefolie zum Einsatz, die ursprünglich für die abdominelle Unterdrucktherapie entwickelt worden ist. Aufgrund ihrer Oberflächenbeschaffenheit ist die Drainagefolie auch für den intrathorakalen Einsatz der Unterdrucktherapie mit Kontakt zum Lungengewebe geeignet. Es handelt sich um die Adaptation der Unterdrucktherapie für die intrathorakale Anwendung beim komplizierten Pleuraempyem. Weitere Studien sind erforderlich, um den zukünftigen Stellenwert der Therapie zu evaluieren.

## Abkürzungen

ANI: Akute Niereninsuffizienz
DE: Drainageelement
ITNPT: Intrathoracic negative pressure therapy
MOV: Multiorganversagen
NPT: Negative pressure therapy
OF: Offenporige doppellagige Drainagefolie
OFPUS: PUS mit OF ummantelt
PE: Pleuraempyem
PUS: Polyurethanschaum
PVA: Polyvinylakohol
THT: Thorakotomie
VATS: Videoassistierte Thorakoskopie
